# Depth-Related Effects on a Meiofaunal Community Dwelling in the Periphyton of a Mesotrophic Lake

**DOI:** 10.1371/journal.pone.0137793

**Published:** 2015-09-09

**Authors:** Bianca Kreuzinger-Janik, Fabian Schroeder, Nabil Majdi, Walter Traunspurger

**Affiliations:** Bielefeld University, Animal Ecology, Konsequenz 45, 33615, Bielefeld, Germany; Université du Québec à Rimouski, CANADA

## Abstract

Periphyton is a complex assemblage of micro- and meiofauna embedded in the organic matrix that coats most submerged substrate in the littoral of lakes. The aim of this study was to better understand the consequences of depth-level fluctuation on a periphytic community. The effects of light and wave disturbance on the development of littoral periphyton were evaluated in Lake Erken (Sweden) using an experimental design that combined *in situ* shading with periphyton depth transfers. Free-living nematodes were a major contributor to the meiofaunal community. Their species composition was therefore used as a proxy to distinguish the contributions of light- and wave-related effects. The periphyton layer was much thicker at a depth of 30 cm than at 200 cm, as indicated by differences in the amounts of organic and phototrophic biomass and meiofaunal and nematode densities. A reduction of the depth-level of periphyton via a transfer from a deep to a shallow location induced rapid positive responses by its algal, meiofaunal, and nematode communities. The slower and weaker negative responses to the reverse transfer were attributed to the potentially higher resilience of periphytic communities to increases in the water level. In the shallow littoral of the lake, shading magnified the effects of phototrophic biomass erosion by waves, as the increased exposure to wave shear stress was not compensated for by an increase in photosynthesis. This finding suggests that benthic primary production will be strongly impeded in the shallow littoral zones of lakes artificially shaded by construction or embankments. However, regardless of the light constraints, an increased exposure to wave action had a generally positive short-term effect on meiofaunal density, by favoring the predominance of species able to anchor themselves to the substrate, especially the Chromadorid nematode *Punctodora ratzeburgensis*.

## Introduction

The littoral zones of lakes are highly dynamic regions subject to important physicochemical fluctuations along horizontal and vertical gradients. In lakes, almost every hard submerged substrate, including stones, woody debris, and macrophytes, is coated by periphyton, defined as a complex community comprising bacteria, fungi, algae, protozoa, and meiofauna embedded in a matrix of exopolymeric substances [1]. The importance of the periphyton community in the functioning of lake ecosystems has been increasingly recognized; for example, periphytic algae have a significant impact on whole-lake primary production [2,3]. Periphyton is also responsible for most of the nitrate depletion in the epilimnion during spring [4] and serves as a habitat and major food resource for many invertebrates and fishes [5,6,7,8,9,10]. Accordingly, periphyton is an important mediator of nutrient cycling in lakes [10,11].

Depth-level fluctuations affect both the magnitude of wave-induced shear stress and the amount of light available for photosynthesis, with important consequences on the structure and functions of periphytic communities [12,13,14]. Because light fuels phototrophic organisms, its limitation is as important as that of nutrients for primary production with upwelling consequences for a variety of consumers [15,16,17]. For periphyton, causes of light limitation include shading effects by riparian vegetation, competition for light with phytoplankton, light absorption by the water column, and self-shading effects within the periphyton mat itself [16]. Several studies have examined the effects of light competition on periphyton by monitoring the responses of its microbial component. A reduction in the amount of light reaching the periphyton was shown to increase the proportion of heterotrophs (bacteria) and/or sciaphilous microphytes [18,19] (reviewed in [20]). However, only a few studies have evaluated the effects of light limitation in a community of metazoan consumers dwelling in the periphyton. In a recent study examining the periphyton of three Swedish lakes, Kazemi-Dinan *et al*. [21] found evidence of a bottom-up-driven shift from the predominance of algivorous towards that of bacterivorous nematodes along a depth gradient. In that study, lake trophic state was a predictor of nematode trophic structure. However, whether the depth-dependent switch in the nematode community structure was due to a bottom-up effect (i.e. light limitation of periphytic algae) or to specific adaptations of algivorous nematodes to the shear stress caused by waves in the shallow zone, or to both, was not resolved. High shear stress reduces periphyton biomass and community structure by mechanical abrasion [22,23]; but in renewing and circulating the water, waves can also stimulate the growth of periphytic algae by improving their access to nutrients [24,25,26]. Wave exposure is known to affect macroinvertebrate and algal communities and thus periphyton functions [13,25,26,27]. By contrast, the impact of wave disturbance on the whole periphyton community, including meiofauna and nematodes, remains to be determined.

The permanent meiofauna comprises minute metazoans (up to 2-mm long; [28]) such as nematodes, rotifers, harpacticoid copepods, and oligochaetes that for most of their life-cycle occupy the benthos. Meiofauna in continental waters has been generally understudied, and in periphytic habitats largely overlooked. Yet periphyton is both a food resource and a suitable habitat for meiofauna. The organisms contributing to the high meiofaunal abundance and diversity in the periphyton are representative of a wide range of feeding strategies that allows exploitation of the diverse microbial resources found in this environment [29,30,31,32]. Because meiofauna is largely unable to evade disturbances of its habitat, investigations of meiofaunal community structure can provide insight into the potential strength of environmental forcing. In addition, the numerous meiofaunal feeding strategies enables studies of the relevance of bottom-up controls. Indeed, in lotic and lentic ecosystems the presence of meiofauna correlates positively with the amount of algae and organic material in the periphyton, consistent with the existence of an important trophic pathway mediated by resident meiofauna [5,33,34,35,36].

This present work aimed at identifying the responses of a periphytic meiofaunal community to depth-related disturbances in a mesotrophic lake. We hypothesized that through increasing light reaching the periphyton: (1) a decrease in water-level might increase phototrophic biomass. Nevertheless, we also expected that (2) this effect might be mitigated by exposure to wave disturbance, through physical erosion of periphytic biomass. Since periphyton is both a habitat and a key food source for meiofauna, (3) we anticipated rather similar responses to effects of water- level fluctuation, although meiofaunacolonizers being mobile, might be more resilient to periphyton erosion than the microphytes embedded in the periphyton.

## Materials and Methods

### Study site

The study was conducted from April to July 2009 in the littoral zone of dimictic Lake Erken, located in central-eastern Sweden (59°51′N, 18°36′E; surface area: 24 km^2^; mean depth: 9 m; max. depth: 21 m). The field sampling was done in agreement with the Erken laboratory, a field station that is a part of the Department of Limnology at the Evolutionary Biology Centre of Uppsala University. No specific permission was required for this lake and no protected species were sampled.

Lake Erken is classified as mesotrophic based on annual mean total phosphorus (P_tot_) and total nitrogen (N_tot_) concentrations of 33 and 787 μg L^−1^, respectively [36]. During the experimental period, there was no ice cover. Nutrient concentrations in the water column did not vary significantly and were similar across the different depths of interest: P_tot_ = 19 and 18 μg L^−1^ and N_tot_ = 730 and 680 μg L^−1^ at 30 and 200 cm depth, respectively. Light intensity was measured underwater using a LI-250A photometer (LI-COR, Lincoln, NE, USA) and showed a typical attenuation profile with increasing depth. At a depth of 620 cm, the amount of light was only 1% of that reaching lake surface. At depths of 30 and 200 cm, the amount of light was 54.8 and 17.4% of that of the surface radiation, respectively.

### Experimental setup

Squared unglazed ceramic tiles with a side length of 4.67 cm and thickness of 0.5 cm were fixed on concrete plates (40 × 40 cm) and used as artificial substrates for periphyton growth. Fifty tiles were placed at a depth of 30 cm and another 50 at a depth of 200 cm. Both sets of tiles were left undisturbed for a period of 10 months prior to the start of the experiment (July 2008–April 2009). To investigate the responses of periphytic communities to a sudden change in depth, the locations of some tiles were switched, such that 15 tiles at a depth of 30 cm (shallow) were transferred to a depth of 200 cm (deep) and 15 tiles at a depth of 200 cm (deep) were transferred to a depth of 30 cm (shallow). These transfers simulated a sudden 170-cm increase or decrease in the water-level of the lake, respectively. To separate the light-induced effects from the wave-induced effects resulting from the transfers, an additional set of tiles (15 from shallow, 15 from deep- to- shallow) was shaded by plastic plates (120 × 100 cm) deployed above the water surface and held in place by metal frames anchored in the ground. This exposed the shallow periphyton to the same light quantity as received at a depth of 200 cm (i.e. 17.4% rather than 54.8% of surface radiation), while leaving exposure to wave disturbances virtually unchanged. In addition to the transferred and shaded tiles, 20 control tiles were left at a depth of 30 cm (shallow) and another 20 control tiles were left at a depth of 200 cm (deep). Thus, our study design consisted of six treatments (shallow, deep, transfer from shallow-to-deep, transfer from deep-to-shallow, shallow-shaded, and transfer from deep-to-shallow-shaded). To monitor the response of periphyton over a relevant period of time, five replicates of each treatment were sampled at 3, 6, and 12 weeks after manipulation and 5 additional replicates were sampled from control tiles at T0 (T0, T3, T6, and T12, respectively).

### Sample analyses

The tiles were carefully removed from the concrete plates, gently placed into plastic bags, and stored at 4°C in the dark until processed a few hours later. The periphyton coating the tiles was completely scraped off using sharp glass slides and then gently mixed in tap water with a blender to obtain a homogeneous suspension [37] used for subsampling in the analyses described below.

To measure the amount of phototrophic organisms and organic matter of the periphyton, two 5- to 15-ml aliquots of the periphyton suspension were filtered onto glass-fiber filters (Whatman GF/C, 25-mm diameter, Whatman, Maidstone, UK). Chlorophyll *a* (Chl *a*), used as an indicator for phototrophic biomass, was extracted by placing one filter in 5–10 ml of acetone for 24 h at 4°C in the dark. Fluorescence quenching was measured spectrophotometrically at 750 and 644 nm and the correspondent concentration of Chl *a* was inferred on the basis of the uncorrected values of pheophytin, following the method of Stich & Brinker [38]. Ash-free dry mass (AFDM) was used as an indicator of total periphytic organic biomass and was measured using a separate filter by the loss-on-ignition method (105°C for 24 h; 530°C for 5h).

The remaining periphyton suspension was preserved with formaldehyde (4%) and stained with Rose Bengal. After filtration of the suspension on 10-μm meshes, invertebrates were counted and identified at a coarse taxonomic level under a dissecting microscope at 40× magnification. Fifty randomly chosen nematodes were sorted from each sample, transferred to glycerol, and mounted on slides following the method of Seinhorst [39]. Nematodes were identified to species level and then classified into different feeding types based on the morphology of their buccal cavity, as proposed by Traunspurger [40]: deposit feeders, epistrate feeders, suction feeders, and chewers. For this study, in total 5000 nematodes were identified corresponding to an average of nearly 25% identified nematodes of the total abundance per sample.

### Data analyses

Differences in periphyton Chl *a*, AFDM, and meiofaunal and nematode density between treatments were assessed using a repeated-measure analysis of variance (rmANOVA). The data were log-transformed to fulfill homogeneity of variances, normally distributed residuals and sphericity (Mauchley test). For the cross-over treatments, a one-way ANOVA for normally distributed data (Bartlett-test) or a Mann-Whitney U-test was used for comparisons between treatments. To test for temporal variations in the response variables, the sampling date was introduced as a covariate in the rmANOVA design. The Spearman rank correlation coefficient was used to measure statistical dependence between response variables.

A non-metric multidimensional scaling (nMDS) based on the Bray-Curtis similarity index was applied to visualize the differences in nematode community structure across the different treatments for each sampling date. nMDS ordination was carried out with square-root-transformed species abundance data, expressed as individuals (ind.) cm^−2^. Distances in nMDS ordination plots are relative because they are based on rank abundances in samples. The dissimilarity between samples is reflected by the relative distances determined from the plot. The quality of the plots is assessed by a stress value, which assumes a good representation of similarities for stress values <0.1 and excellent representations for stress values <0.05.

A permutational multivariate analysis of variance (PERMANOVA) was used to test for differences in nematode community structure across treatments at each sampling date.

Statistica software (version 9.1; StatSoft, Tulsa, OK, USA) was used for the rmANOVA and Spearman correlations. Community analyses were carried out using the PRIMER v6.1.13 software package with PERMANOVA+ add-on v1.0.3 (PRIMER-E, Plymouth, UK).

## Results

### Periphyton

#### Temporal and vertical distribution

**Fig 1:**
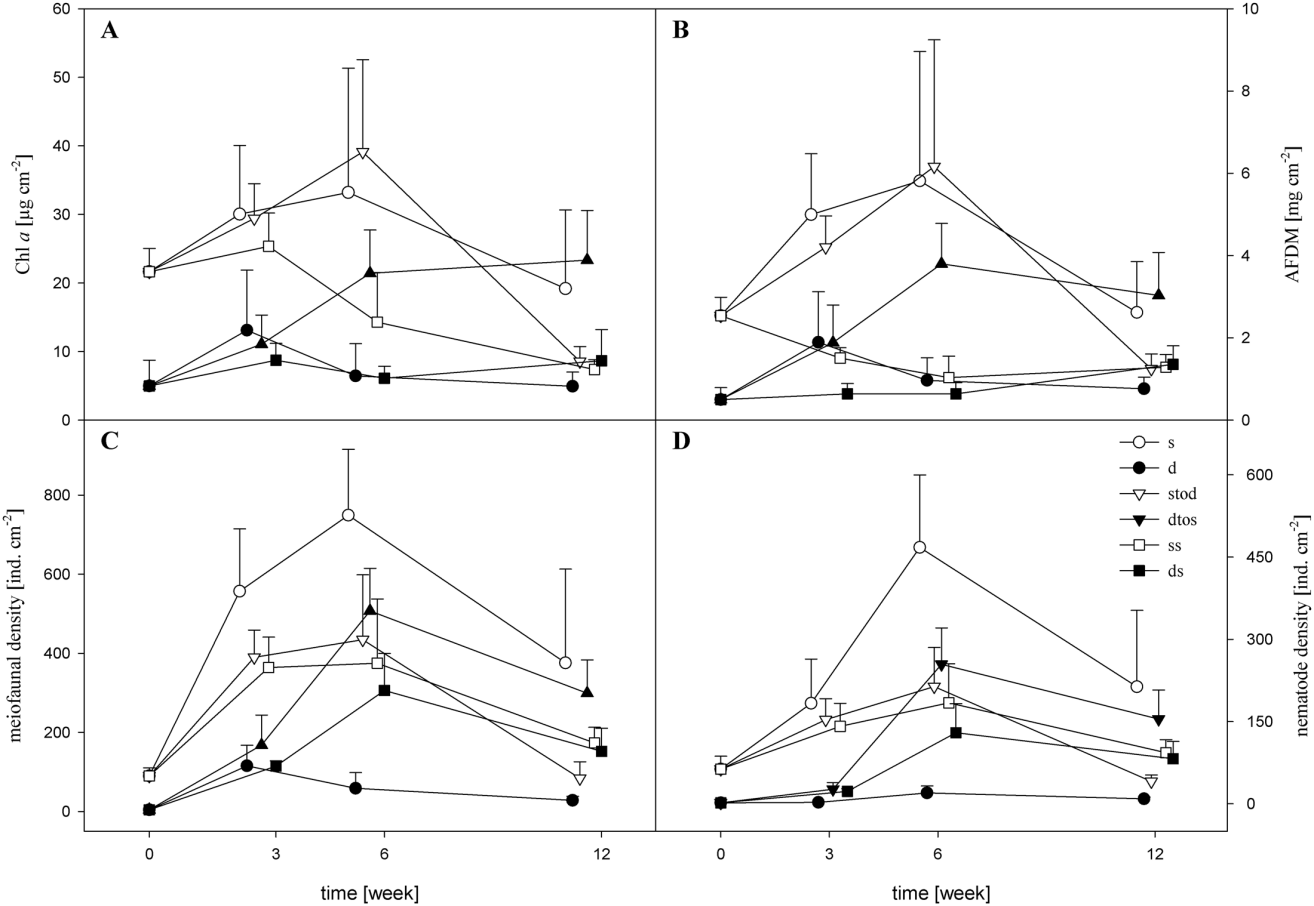
Dynamics of (a) phototrophic biomass measured as chlorophyll a (Chl *a*) concentration, (b) organic matter content measured as ash-free dry mass (AFDM), (c) meiofaunal density, and (d) nematode density in the periphyton of Lake Erken. Data (mean, n = 5, +SD) are given for six different depth treatments (s: shallow, d: deep, s to d: shallow-to-deep, d to s: deep-to-shallow, ss: shallow-shaded, and ds: deep-to-shallow-shaded).

The Chl *a* concentration in the periphyton increased in all treatments beginning at week 3 of the experiment (Fig 1a). The amount of periphytic Chl *a* was significantly higher in the shallow than in the deep treatments (rmANOVA, F_1,21_ = 47.4, p< 0.001) and differed over time (F_3,21_ = 3.6, p< 0.05), whereas the interaction between depth and time was not significant. The Chl *a* concentration was three- to four-times higher at a depth of 30 cm than at a depth of 200 cm, with significant depth-dependent differences recorded at T0, T6, and T12 (rmANOVA, T0: p < 0.01; T6: p < 0.001; T12: p < 0.05).

The variation in the organic content of the periphyton (AFDM) during the experiment is shown in Fig 1b. Similar to Chl *a* levels, the amount of periphytic AFDM was significantly higher in the shallow than in the deep treatment (rmANOVA, F_1,21_ = 47, p < 0.001) and differed over time (F_3,21_ = 8, p < 0.001), whereas the interaction between depth and time was not significant. Generally, the pattern of AFDM resembled that of Chl *a*, with significant differences between shallow and deep treatments recorded across all sampling dates (rmANOVA, T0: p < 0.01; T3: p < 0.05; T6: p < 0.001; T12: p < 0.05).

#### Effects of depth transfer

Chl *a* and AFDM differed significantly over time in the two cross-over treatments (Table 1). In the shallow-to-deep treatment, the Chl *a* concentration reached a peak of 39.1 μg cm^−2^ after 6 weeks before falling to 8.6 μg cm^−2^ (Fig 1a). A comparison of the Chl *a* concentration between the shallow-to-deep and deep treatments showed significant higher values for shallow-to-deep until week 6 (Mann-Whitney U-test, p < 0.05) whereas after 12 weeks the values were similar. Chl *a* in the deep-to-shallow treatment increased steadily to 23.4 μg cm^−2^. The difference compared to the shallow treatment was significant only after 3 weeks (Mann-Whitney U- test, p < 0.01).

**Table 1:** Summary of rmANOVA of temporal differences in the amounts of periphytic chlorophyll a (Chl *a*) and AFDM and in meiofaunal and nematode densities.

	Chl *a*				AFDM				meiofauna				nematodes			
Effects	df	MS	*F*	*p*	df	MS	*F*	*p*	df	MS	*F*	*p*	df	MS	*F*	*p*
s to d	3	0.41	37.7	***	3	0.2	25.0	***	3	1.4	77.6	***	3	0.5	40.6	***
d to s	3	0.59	16.3	***	3	0.3	20.1	***	3	5.4	120.4	***	3	4.0	120.0	***
ss	3	0.3	9.1	**	3	0.1	9.6	**	3	1.5	50.5	***	3	0.2	6.7	**
ds	3	0.08	1.3	n.s.	3	0.0	3.8	n.s.	3	3.4	154.4	***	3	2.1	82.5	***

Cross-over and shaded treatments (shallow-to-deep: s to d, deep-to-shallow: d to s, shallow-shaded: ss, and deep-shaded: ds) were set as independent factors with four temporal modalities. Significance level (*p*): n.s.: not significant,

**: significant at *p* < 0.01,

***: significant at *p* < 0.001.

The temporal pattern of shallow-to-deep AFDM was generally the same as that of the shallow treatment, with significant higher values compared to the deep treatment recorded until week 12 (one-way ANOVA, T0: F_1,8_ = 72.01, p < 0.001; T3: F_1,7_ = 11.57, p < 0.05; T6: F_1,8_ = 35.14, p < 0.001). However, large increases occurred in the deep-to-shallow AFDM such that after 6 and 12 weeks the organic matter content was similar to that of the non-transferred shallow periphyton (Fig 1b). The differences after 3 weeks were significant (one-way ANOVA, F_1,8_ = 15.64, p< 0.01) but after 6 and 12 weeks they were not. Thus, the positive response of algal and organic biomass following transfer of the deep periphyton to a shallow location (i.e. simulating a decrease in the lake's water level) occurred much more rapidly than the development of a negative response to the transfer to a deep location (i.e. simulating an increase in the lake's water level).

#### Effects of wave disturbance

In the shallow-shaded treatment, Chl *a* and AFDM varied significantly within the experimental period while in the deep-shaded treatment no temporal variation was observed (Table 1). Over the 12 weeks of the experiment, Chl *a* values decreased steadily in the shallow-shaded treatment, reaching a minimum of 7.4 μg cm^−2^ (Fig 1a), whereas they were two- to three-fold higher in the shallow and deep-to-shallow treatments. In the shallow-shaded and the shallow-to-deep treatments, Chl *a* concentrations at T12 were very similar; however, this was likely due to the abrupt reduction of Chl *a* in the latter treatment between T6 and T12, since previously the Chl *a* concentration had been much higher in the shallow-to-deep than in the shallow-shaded treatment (one-way ANOVA, T6: F_1,8_ = 226.7, p < 0.001). Chl *a* concentrations in the deep-to-shallow-shaded (deep-shaded) and deep treatments did not differ significantly (Fig 1a).

In the shallow-shaded treatment, AFDM decreased steadily during the first 6 weeks (Fig 1b), resulting in highly significant lower values compared to the shallow-to-deep treatment, after 3 weeks (one- way ANOVA: F_1,8_ = 88.38, p < 0.001) and 6 weeks (F_1,8_ = 32.63, p < 0.001). Thus, regardless of light limitations, the higher exposure to wave disturbance resulted in short-term decreases in periphyton biomass. In the deep-to-shallow-shaded treatment, AFDM levels were low and did not significantly differ from those of the deep treatment.

### Meiofauna

#### Composition and distribution

**Fig 2:**
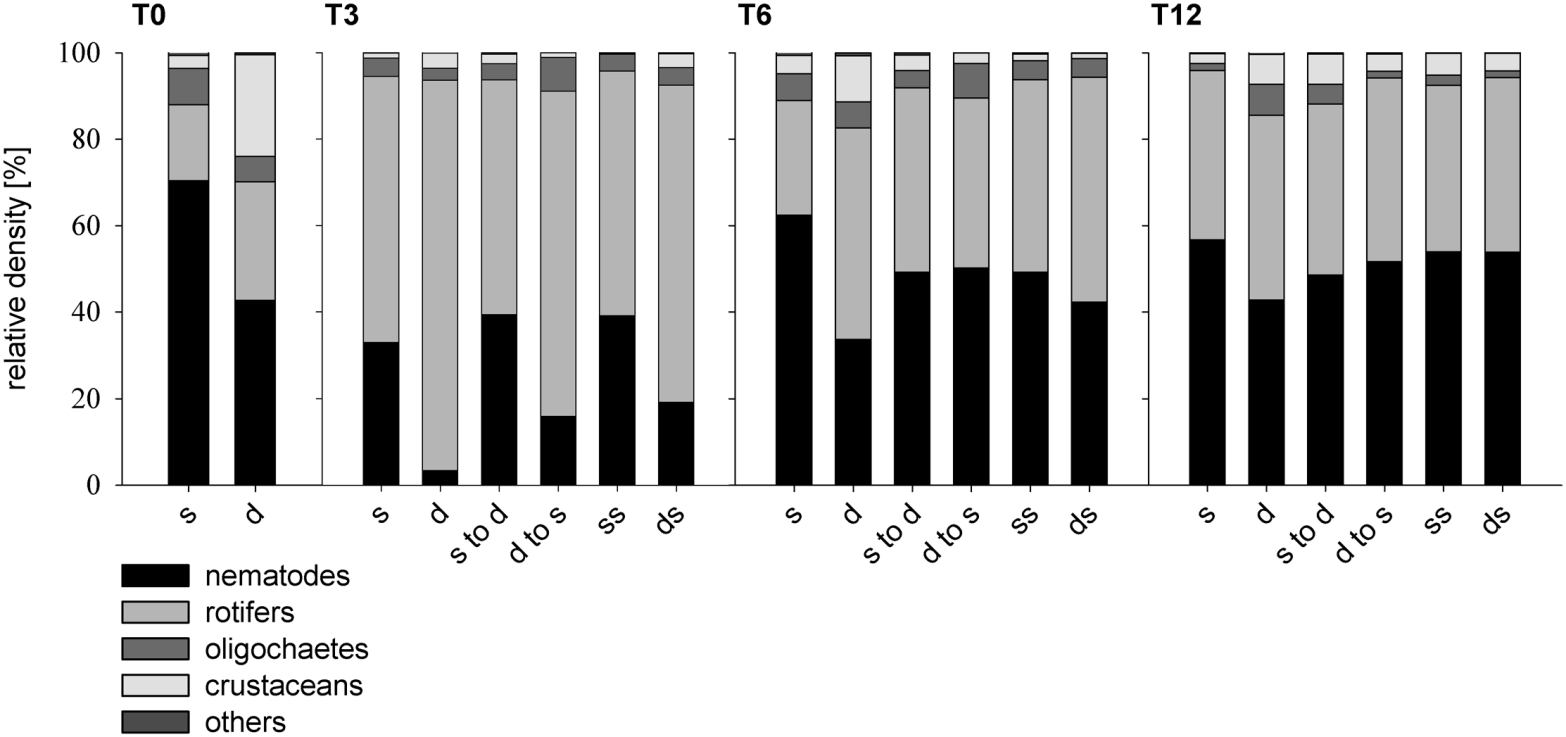
Relative meiofaunal density. Densities (mean, N = 5) of nematodes, rotifers, oligochaetes, micro-crustaceans (copepods, cladocerans, and ostracodes) and other meiofauna (water mites and tardigrades) in the periphyton of Lake Erken in response to six different depth treatments (shallow: s, deep: d, shallow-to-deep: s to d, deep-to-shallow: d to s, shallow-shaded: ss and deep-shaded: ds) at four sampling dates (T0: start, T3: 3 weeks, T6: 6 weeks, and T12: 12 weeks).

The temporal dynamics of the density of periphytic meiofauna (Fig 1c) showed important population fluctuations. Meiofaunal densities ranged from < 5 to >750 ind. cm^−2^ and the correlations with Chl *a* and AFDM were positive and highly significant (Spearman; ρ > 0.687; p < 0.001). Eight meiofaunal groups were found in the periphyton: nematodes, rotifers, oligochaetes, harpacticoid copepods, cladocerans, ostracods, tardigrades, and water mites (Fig 2). Generally nematodes dominated, although, in deeper periphyton, the contributions of micro-crustaceans (harpacticoids, cladocerans and ostracods) and rotifers to the meiofaunal community were larger than the contribution of nematodes (Fig 2). Overall, meiofaunal density differed significantly both between shallow and deep treatments (rmANOVA, F_1,21_ = 374, p <0.001) and over time (F_3,21_ = 53, p < 0.001). The interactions between depth and time (F_4,21_ = 0.2, p < 0.05) were significant, as meiofaunal density was up to one-order of magnitude higher in shallow than in deep periphyton, with highly significant differences measured at all sampling dates (rmANOVA, p < 0.001).

#### Effects of depth transfer

Meiofaunal density also differed significantly over time in the two cross-over treatments (Table 1), reaching a peak in the shallow-to-deep treatment of 450 ind. cm^−2^ after 6 weeks before falling almost to the initial density of 84 ind. cm^−2^ after 12 weeks (Fig 1c). A comparison of meiofaunal density in the shallow-to-deep vs. deep treatments showed a significant higher density in the shallow-to-deep treatment until week 6 (T0: Mann- Whitney U-test: p < 0.01; one- way ANOVA, T3: F_1,7_ = 16.9, p< 0.01; T6: F_1,8_ = 37.92, p < 0.001), but not at week 12. Large increases in meiofaunal density occurred in the deep-to-shallow treatment during the first 6 weeks, peaking at 507 ind. cm^−2^. The differences compared to the shallow treatment at weeks 3 and 6 were significant (one- way ANOVA, T3: F_1,7_ = 54.85, p < 0.001; T6: F_1,8_ = 7.34, p < 0.05).

#### Effects of wave disturbance

Meiofaunal density was significantly affected by shading over time (Table 1) such that at week 12 it was two-fold lower in the shallow-shaded than in the shallow and deep-to-shallow treatments (Fig 1c). In contrast to Chl *a* and AFDM, meiofaunal density in the deep-to-shallow-shaded (deep-shaded) treatment increased steadily, reaching 306 ind. cm^−2^ after 6 weeks. The meiofaunal density was significantly higher in the deep-shaded than in the deep treatment after 6 and 12 weeks (one- way ANOVA, T6: F_1,8_ = 26.54, p < 0.001; T12: F_1,6_ = 48.93, p < 0.001), indicating a positive response of meiofaunal density to decreasing depth that was not mediated by either increasing light or an increase in the availability of potential periphytic resources.

### Nematodes

#### Density distribution

Nematode density fluctuated greatly, ranging from 2 to 468 ind. cm^−2^ (Fig 1d), and correlated positively with Chl *a* and AFDM (Spearman; ρ > 0.697; p < 0.001). Nematodes are a major component of meiofauna and their responses to depth transfer and shading were similar (Table 1 and Fig 1c and 1d). The exception was the interaction between time and depth, as the differences in nematode density between the shallow and deep treatments were not significant (p = 0.06). Moreover, nematode density continued to increase 6 weeks after the transfer from shallow to deep water and reached a peak of 213 ind. cm^−2^, which was ten times higher than the density measured in non-transferred deep periphyton. The nematode density was significantly higher in shallow-to-deep treatment than in the deep treatment at all sampling dates (one- way ANOVA, T0: F_1,8_ = 150.37, p < 0.001; T3: F_1,7_ = 390.44, p < 0.001; T6: F_1,8_ = 60.31, p < 0.001; T12: F_1,7_ = 52.99, p < 0.001). Nematode density in the deep-to-shallow treatment was lower than in the shallow treatment until week 6 (one- way ANOVA, T0: F_1,8_ = 150.37, p < 0.001; T3: F_1,8_ = 35.8, p < 0.001; T6: F_1,8_ = 10.73, p < 0.05), but after 12 weeks similar densities were recorded. Nematode density in the shaded treatments followed the same pattern as that of the meiofauna (Fig 1c and 1d) and was much higher in the deep-to-shallow-shaded than in the deep treatment. The differences measured after 6 and 12 weeks were significant (one-way ANOVA, T6: F_1,8_ = 32.28, p < 0.001; T12: F_1,6_ = 77.85, p < 0.001). Among the meiofauna, nematodes benefited rapidly from an increased exposure to wave action, as their density increased regardless of light and resource constraints.

#### Community structure

Nineteen nematode species were found from a total of 4850 nematode individuals identified. Among the 19 nematode species, two species, *Punctodora ratzeburgensis* and *Chromadorina viridis*, were present in every sample (S1 Table). The community was largely dominated by the epistrate feeder *P*. *ratzeburgensis*, which accounted for > 80% of the nematode community in shallow periphyton. After 12 weeks, C. *viridis* was co- dominant with *P*. *ratzeburgensis*. In general, the relative abundance of *C*. *viridis* was highest in the deep and shallow-to-deep treatments between T0 and T12. At T0 and T3, *Eumonhystera vulgaris* was the second most abundant species, after *P*. *ratzeburgensis* (except in the shallow and shallow-to-deep treatments). The genus *Eumonhystera*, represented by five other species in addition to *E*. *vulgaris*, comprised approximately one-third of all identified nematodes (S1 Table).

Ten nematode species belonged to the feeding type of deposit feeders, while suction feeders and chewers appeared only sporadically. The family Tobrilidae was represented by three species and was therefore the most common predatory/omnivorous (chewer) species. The relative occurrences of deposit and epistrate feeders correlated positively with the amounts of phototrophic and organic biomass in the periphyton (Table 2).

**Table 2:** Spearman correlation coefficient between feeding- type and Chl *a* and AFDM content.

Feeding-type	Chl *a*	AFDM
Deposit-feeders	0.734 ***	0.683 ***
Epistrate-feeders	0.694 ***	0.685 ***
Suction-feeders	0.193 **	0.169 n.s.
Chewers	-0.11 n.s.	-0.062 n.s.

ρ testing the statistical dependence of the relative occurrence of nematode feeding types on the amount of chlorophyll *a* (Chl *a*) and ash-free dry (organic) material (AFDM) in the periphyton of Lake Erken. Significance level (*p*): n.s.: not significant,

**p < 0.01,

***p < 0.001.

After 6 weeks, species richness (12 species) was highest in the shallow-to-deep treatment (S1 Table) and lowest in the deep-to-shallow-shaded treatment, sharply decreasing from nine to only three different species (*P*. *ratzeburgensis*: 95.7%; *C*. *viridis*: 2.4%; *Plectus tenuis*: 1.9%; S1 Table) 12 weeks after the transfer. As a general pattern, nematode species richness was higher in deep than in shallow periphyton.

**Fig 3:**
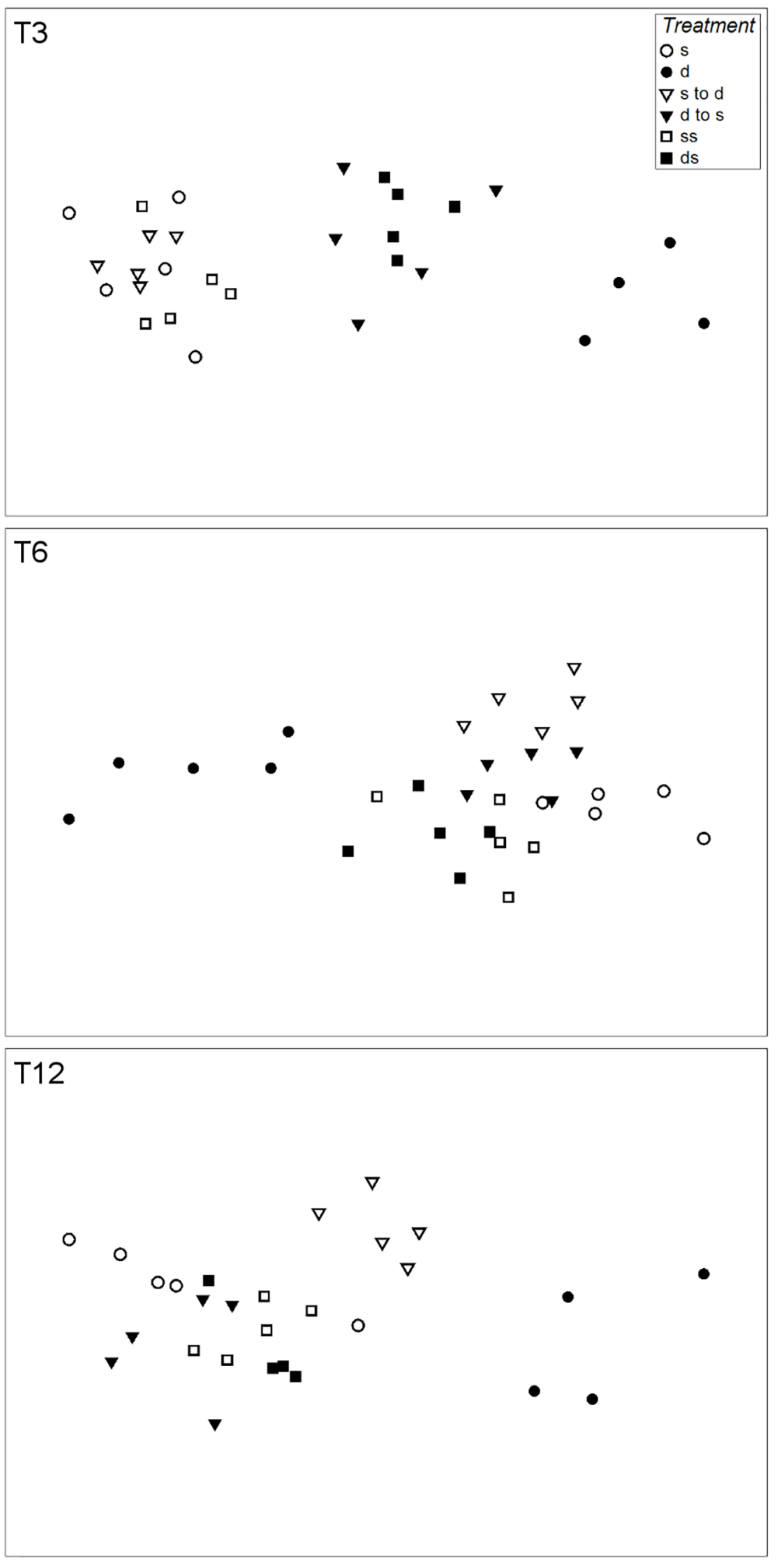
Non-metric multidimensional scaling (nMDS) plot of nematode species composition. Data is shown for samples retrieved after 3, 6, and 12 weeks (T3, T6, and T12, respectively). The nMDS calculations were based on the Bray- Curtis similarity with square root- transformed nematode density data (ind. cm^−2^) as determined in the epilithon of Lake Erken. Stress values: T3 = 0.07; T6 = 0.11; T12 = 0.09.

At all sampling dates, the nematode community in the deep treatment could be clearly distinguished from that in the other treatments (Fig 3, Table 3). Significant differences in the structure of the nematode community were determined among the treatments at all dates (Fig 3, Table 3). After 3 weeks, the nematode communities in the deep-to-shallow and in the deep-shaded clustered together as did those in the shallow, shallow-to-deep, and shallow-shaded treatments. After 6 weeks, the two shaded treatments showed higher similarity and clustered together with the cross-over treatments. At the last sampling date, the shallow-to-deep treatment was clearly segregated from the other treatments (Fig 3, Table 3).

**Table 3:** PERMANOVA.

	T3	T6	T12
factor groups	*p*	*p*	*p*
Global	***	***	***
s vs. d	**	**	*
s vs. s to d	n.s.	**	*
s vs. d to s	**	*	n.s.
s vs. ss	n.s.	*	n.s.
s vs. ds	**	*	n.s.
d vs. s to d	*	**	*
d vs. d to s	**	*	**
d vs. ss	**	*	*
d vs. ds	**	**	*
s to d vs. d to s	*	**	**
s to d vs. ss	**	**	**
s to d vs. ds	*	*	**
d to s vs. ss	**	*	n.s.
d to s vs. ds	n.s.	**	*
ss vs. ds	**	n.s.	n.s.

PERMANOVA testing the statistically significant clustering of all treatments (shallow-to-deep: s to d, deep-to-shallow: d to s, shallow-shaded: ss and deep-shaded: ds). Significance level (*p*): n.s.: not significant,

*p < 0.05,

**p < 0.01,

***p < 0.001.

The community structure of nematodes transferred from deep to shallow water differed from that of nematodes in the shallow treatment at T3 but was similar thereafter (Table 3). High structural similarities in the nematode community were also observed in the shallow-shaded, deep-to-shallow, and deep-shaded treatments from week 6 onwards, based on the very strong dominance (>95%) of *P*. *ratzeburgensis* in samples (S1 Table).

## Discussion

Our results clearly show that even a relatively small fluctuation (170 cm) in the water level of a lake can have important consequences on the abundance and composition of its littoral periphytic community. After 10 months of incubation, the periphyton was much more substantial in the shallow (30 cm) than in the deeper (200 cm) depth, based on higher organic and phototrophic biomasses and the higher meiofaunal and nematode densities. Conversely, a greater diversity and a higher similarity characterized the meiofaunal and nematode communities in the deep zone (200 cm depth). In the following, we discuss the effects of depth fluctuations on a periphytic community and its response to changes in its exposure to light and wave conditions.

### Periphyton

Periphyton transfer from a deep to a shallow zone of the lake resulted in rapid increases in periphytic algal and organic biomass towards the values determined in non-transferred shallow periphyton. However, transfer from a shallow to a deep zone did not cause a decline to the low biomass values consistently observed in the periphyton incubated in the deep zone. In fact, the biomass dynamics of periphyton transferred from a shallow to a deep zone were fairly similar to those of the non-transferred shallow periphyton. This finding suggests that a decrease in depth level initiates a rapid, positive response by primary producers in the periphyton, whereas a negative response following water level increase is buffered by a resilience mechanism. Periphyton biomass is stimulated by light (e.g. [15,41,42]), as evidenced by the increase in biomass attributable to photosynthesis [16]. Thus, with increasing water depth primary production by periphyton decreases [17] because the attenuated light lowers algal growth rates even if there is little change in the nutrient supply between shallow and deep water (as was the case in our study). Self-shading effects within the mat can also reduce the availability of light to basal layers, thus favouring sciaphilous microphytes in basal periphytic layers [20]. These microphytes were likely to have been already present in the periphyton transferred from shallow to deep zones, which could explain the mechanism of higher resilience of phototrophic biomass to water level increase.

Light was not the only constraint affecting periphytic biomass. Comparisons between deep-to-shallow and deep-to-shallow-shaded treatments showed that, in the absence of stimulation by increasing light availability, a transfer to the shallow zone did not result in significant biomass accrual. Comparisons between shallow-to-deep and shallow-shaded treatments further suggested that at comparable light levels the higher exposure to wave disturbance in the shallow zone negatively affected periphytic biomass, with very little latency. After a 10-month habituation period to wave-exposure conditions, sudden shading triggered stronger effects on the thick layer of periphyton that had developed in the shallow zone than on the thin layer of periphyton that had formed in the deep zone. The effects of shear stress on periphyton were shown to depend on the thickness [22,25], and the degree of senescence of the algal layers of the periphyton [43]. According to our results, the consequences of shading on the periphyton in the shallow littoral zones of lakes are likely to be magnified by shear stress [23,44]. This finding raises questions regarding the potentially strong impediment of benthic primary production in shallow littoral zones of lakes artificially shaded by construction or embankments.

### Meiofauna

The response of meiofauna to a depth transfer was very similar to that of the phototrophic and organic biomass of the periphyton, except that the positive response of meiofauna transferred from deep to shallow water was slightly slower than that of phototrophic and total organic biomass, occurring at T6 rather than T3. Meiofauna and nematode density dynamics were similar between deep-to-shallow and deep-to-shallow-shaded treatments, as well as between shallow-to-deep and shallow-shaded treatments (Fig 1c and 1d). These findings suggested that compared to periphytic biomass, meiofaunal consumers were less affected by wave disturbance. Waves can detach parts of the mat [23,45], resulting in free-floating mat pieces that can harbor high densities of nematode juveniles and gravid females [34]. Peters et al. [46] showed that, in lakes, mat dispersal via water-column transport was the main pathway of periphyton colonization by meiofauna. The suspension of an object in the water column depends on the local magnitude of the shear stress [47]; thus, in the case of sloughing periphyton, erosion or drift can allow meiofauna to quickly colonize available habitats. This is especially the case in shallow areas, where because of higher mixing by wave action, the pool of ‘suspended’ meiofauna colonizers should be important.

Meiofaunal responses to the different depth conditions examined in this study varied. Thus, rotifers markedly dominated in the non-transferred deep periphyton and, after 3 weeks, in the deep-to-shallow transfer. Between weeks 3 and 12, the meiofaunal community was very similar to that of the non-transferred shallow periphyton, where nematodes dominated consistently. The temporary dominance of rotifers in the early stages of periphyton colonization in lakes and rivers process has been reported [33,46,48]: In their *in situ* colonization experiment in Lake Constance, Peters et al. [46] found that between days 2 and 29 of colonization, rotifers were the most abundant group at a depth of 70–90 cm. The dominance of rotifers during the early phase of colonization is consistent with the short-life cycle of these fully parthenogenetic organisms, their well-developed ciliature that facilitates swimming, and their ability to enter a resting state to cope with habitat disturbances [49].

Chl *a* and AFDM availabilities were tightly coupled with the density of periphytic meiofauna. As amply suggested in the literature [5,21,33,34,35,36,50], these relationships highlight the importance of bottom-up controls in structuring the meiofaunal community inhabiting the periphyton. However, little is known about the feeding preferences of meiofauna in continental waters (but see [21,31,51,52]). Our results suggest that, although there was less habitat space in the deeper periphyton, the higher group diversity there could match with a shift in microbial community structure from predominance of phototrophic towards that of heterotrophic microbes. The plasticity of the transferred meiofauna with respect to the different light conditions also suggests an ability to rapidly switch to heterotrophic resources when a sudden reduction in the amount of light hinders primary production.

### Nematodes

In our study, nematodes were important contributors to the meiofaunal community, in agreement with evidence from a variety of other freshwater benthic ecosystems [36,53,54,55]. Schroeder et al. [32] reported densities >200 ind. cm^−2^ in periphyton at a depth of 50 cm in Lake Erken from April to August 2009, including peak nematode densities over 1000 ind. cm^−2^ in June 2009. Such huge nematode densities were also determined in the present work, peaking after 6 weeks (i.e. in June 2009) in all treatments. However, the densities were much higher in shallow than in deep periphyton (467.7 vs. < 20 ind. cm^−2^). By contrast, in the periphyton of neighboring eutrophic Lake Limmaren, nematode densities at depths of 150 and 50 cm were 316.4 and 23.4 ind. cm^−2^, respectively [21]. The authors of the latter study suggested that the wave action in the shallow zones of eutrophic lakes may restrict the amount of sinking organic material available to benthic consumers whereas deeper water provides a more stable depositional zone. According to our results, this is not the case in mesotrophic Lake Erken, since wave action did not significantly alter the colonization of periphyton by nematodes as long as sufficient phototrophic biomass was available.

Compared to freshwater sediments, there was little diversity in the nematode community of the periphyton (19 species), which throughout the study was strongly dominated by an assemblage of only a few species. The epistrate feeder *Punctodora ratzeburgensis* (family Chromadoridae) accounted for > 80% of the nematode community across all samples except in the non-transferred deep periphyton and in some of the shallow-to-deep periphyton samples. Although temporal fluctuations can occur in the short-term, the strong dominance of one chromadorid species in lotic and lentic periphytic habitats appears to be commonplace [5,21,29,32,34,35,56] and could be ascribed to the highly unstable nature of periphytic habitats. In fact, periphyton is a self-produced habitat positioned at the interface with the water column, such that it is greatly affected by abiotic (e.g., light, shear stress) and biotic (e.g., production, grazing, bioturbation) controls. Within this environment, a few species may be able to outcompete others, based on their successful adaptations to a periphytic life-style. Chromadorid nematodes have adapted to shear stress by being able to attach themselves to the substrate surface using sticky secretions from their caudal gland [57,58]. These nematodes also graze on periphytic diatoms by puncturing or cracking their frustules [31,59,60,61]. These features make chromadorid nematodes competitive colonizers of periphytic habitats, such that the establishment of many other nematode species could be prevented, in accordance with the competitive exclusion principle [62]. In their monitoring of Lake Erken, Schroeder et al. [32] found that the population dynamics of *P*. *ratzeburgensis* in periphyton in the spring–summer 2009 were characterized by a bloom event that occurred during or directly after the clear water stage of the lake. The period of our study included this bloom event. Its consequences were evident by the trend towards a homogenization of the nematode community structure in all samples from April to July 2009.

Despite the reduced amount of habitat space, the non-transferred deep periphyton harbored greater diversity and a lower proportion of chromadorids. Instead, bacterial-feeding species such as *Eumonhystera vulgaris* contributed up to one third of the nematode assemblage found in deep periphyton, The influence of shear stress decreases with increasing depth, and therefore so does the advantage of chromadorids over other nematode species. Deep periphyton may also contain a larger proportion of bacteria that decompose organic detritus, which may increase the range of trophic niches available for bacterial-feeding nematodes such as *Eumonhystera* spp. [21,63]. The structure of the nematode community at 200 cm depth was clearly different from that of all other treatments, indicating that depth exerts long-term effects on nematode community composition. When transferred to the shallow zone, the previously deep nematode community changed quickly such that in only 3 weeks it resembled that of the non-transferred shallow periphyton. A reverse adjustment (shallow-to-deep) was not observed even at 12 weeks after the transfer, suggesting that a sudden decrease in lake water level may trigger larger effects on the periphytic nematode community than a sudden increase in lake water level.

The relative influences of waves and light on nematode community structure could not be easily distinguished because the strong dominance of *Punctodora ratzeburgensis* in all shaded or transferred treatments hampered the detection of subtle changes therein. However, our results further demonstrate the ability of *P*. *ratzeburgensis* to quickly colonize and overwhelm nematode communities in the periphyton of Lake Erken. In particular, regardless of light constraints, wave exposure strongly affected nematode community structure, as it differed significantly between the shallow-shaded and shallow-to-deep treatments. Moreover, the dissimilarity increased with increasing incubation time. The significant differences in the nematode community between the non-transferred deep periphyton and the deep-to-shallow-shaded periphyton were more quantitative than qualitative and could be explained by the positive effect of wave disturbance on the density of *P*. *ratzeburgensis*, the dominant nematode species dwelling the periphyton of lake Erken.

## Supporting Information

S1 Table Mean relative abundance (%) and species richness (S) of nematode species in the littoral zone of Lake Erken. Data (n = 5) are given for all treatments (shallow-to-deep: s to d, deep-to-shallow: d to s, shallow-shaded: ss and deep-shaded: ds) at all sampling dates (T0: start, T3: 3 weeks, T6: 6 weeks, and T12: 12 weeks). The feeding types (FTs) were classified following the method of Traunspurger [40]: deposit feeder (D), epistrate feeder (E), suction feeder (S), and chewer (C).
(DOCX)
