# Supplementary material for: Depth-Related Effects on a Meiofaunal Community Dwelling in the Periphyton of a Mesotrophic Lake
Source: PLoS One. 2015 Sep 9;10(9):e0137793. doi: 10.1371/journal.pone.0137793 (PMC4564161; doi:10.1371/journal.pone.0137793)
Supplement: S1 Table — Data (n = 5) are given for all treatments (shallow-to-deep: s to d, deep-to-shallow: d to s, shallow-shaded: ss and deep-shaded: ds) at all sampling dates (T0: start, T3: 3 weeks, T6: 6 weeks, and T12: 12 weeks). The feeding types (FTs) were classified following the method of Traunspurger [40]: deposit feeder (D), epistrate feeder (E), suction feeder (S), and chewer (C). (DOCX) [file pone.0137793.s001.docx]

|  |  | T0 | | T3 | | | | | | T6 | | | | | | T12 | | | | | |
| --- | --- | --- | --- | --- | --- | --- | --- | --- | --- | --- | --- | --- | --- | --- | --- | --- | --- | --- | --- | --- | --- |
| nematode taxa | FT | s | d | s | d | s to d | d to s | ss | ds | s | d | s to d | d to s | ss | ds | s | d | s to d | d to s | ss | ds |
| *Eumonhystera barbata*  Andrassy, 1981 | D | 2.4 | - | 0.5 | 6.4 | - | 0.3 | 1.0 | 0.9 | - | 0.2 | 2.9 | 0.5 | - | 0.4 | - | 0.1 | 0.3 | - | - | - |
| *Eumonhystera dispar*  Bastian, 1865 | D | 0.5 | - | - | - | - | 1.0 | 1.6 | 0.5 | 0.3 | - | 0.8 | 1.0 | 0.5 | 0.4 | 0.3 | 0.3 | - | 0.3 | 1.0 | - |
| *Eumonhystera filiformis*  Bastian, 1865 | D | - | 2.8 | - | - | - | - | - | - | - | 1.3 | - | - | - | - | - | - | - | - | - | - |
| *Eumonhystera pseudobulbosa*  von Daday, 1896 | D | 0.5 | 11.9 | - | 0.5 | 0.3 | - | - | - | - | - | 2.0 | 1.7 | - | - | - | - | - | - | - | - |
| *Eumonhystera simplex*  de Man, 1880 | D | - | - | - | 1.0 | 0.3 | - | - | - | - | - | - | 0.4 | - | - | - | - | - | - | - | - |
| *Eumonhystera vulgaris*  de Man, 1880 | D | 10.3 | 31.1 | 2.7 | 23.6 | 7.4 | 6.9 | 7.8 | 4.3 | 3.5 | 18.6 | 9.8 | 2.6 | 0.8 | 0.7 | - | 0.5 | - | - | 0.3 | - |
| *Monhystera* spec. | D | 0.3 | 0.7 | - | - | - | - | - | - | - | - | - | - | - | - | - | 0.1 | - | - | - | - |
| *Plectus aquatilis*  Andrassy, 1985 | D | - | 1.0 | 0.3 | 0.5 | - | 0.7 | - | 0.5 | 1.1 | - | 0.4 | - | - | - | - | - | - | 1.0 | - | - |
| *Plectus parvus*  Bastian, 1865 | D | - | - | - | - | - | - | - | - | - | - | - | - | 0.4 | - | - | - | - | - | - | - |
| *Plectus tenuis*  Bastian, 1865 | D | 1.4 | 0.7 | 1.1 | 1.7 | 2.0 | - | - | 0.5 | 2.7 | - | 1.3 | - | 1.3 | 0.8 | 1.8 | 1.4 | 0.4 | 0.4 | 0.9 | 1.9 |
| *Chromadorina viridis*  von Linstow, 1876 | E | 2.4 | 6.5 | 5.5 | 8.3 | 8.8 | 2.6 | 2.6 | 1.1 | 3.4 | 9.3 | 9.7 | 2.2 | 3.6 | 2.2 | 2.9 | 3.4 | 8.2 | 0.7 | 1.3 | 2.36 |
| *Chromadorita leuckarti*  de Man, 1876 | E | - | 0.7 | - | 1.3 | - | - | - | - | - | - | 0.4 | - | 0.4 | - | - | 0.2 | 1.4 | - | - | - |
| *Punctodora ratzeburgensis*  von Listow, 1876 | E | 81.3 | 43.8 | 89.3 | 54.7 | 80.7 | 86.7 | 87.0 | 91.6 | 88.0 | 70.0 | 71.2 | 91.2 | 93.0 | 95.6 | 94.7 | 93.2 | 85.1 | 97.2 | 96.2 | 95.7 |
| *Crocodorylaimus flavomaculatus*  von Linstow, 1876 | S | 0.9 | - | - | - | - | - | - | 0.3 | 1.0 | - | 0.4 | 0.4 | - | - | 0.3 | 0.4 | 0.9 | 0.3 | 0.4 | - |
| *Dorylaimus stagnalis*  Dujardin, 1845 | S | - | - | - | - | - | 0.1 | - | - | - | - | - | - | - | - | - | - | - | 0.3 | - | - |
| *Ironus tenuicaudatus*  de Man, 1876 | C | - | - | - | - | 0.6 | 0.5 | - | - | - | 0.6 | 0.6 | - | - | - | - | 0.2 | 3.0 | - | - | - |
| *Tobrilus gracilis*  Bastian, 1865 | C | - | - | 0.3 | - | - | - | - | - | - | - | 0.4 | - | - | - | - | 0.2 | - | - | - | - |
| *Semitobrilus cf pellucidus*  Bastian, 1865 | C | - | - | 0.3 | 2.0 | - | 1.3 | - | 0.5 | - | - | - | - | - | - | - | - | - | - | - | - |
| *Brevitobrilus stefanskii*  Micoletzkey, 1925 | C | - | 0.7 | - | - | - | - | - | - | - | - | - | - | - | - | - | - | - | - | - | - |
| *Tripyla glomerans*  Bastian, 1865 | C | - | - | - | - | - | - | - | - | - | - | - | - | - | - | - | - | 0.7 | - | - | - |
| species richness |  | 6 | 10 | 8 | 10 | 6 | 9 | 5 | 9 | 7 | 6 | 12 | 7 | 7 | 6 | 5 | 11 | 8 | 8 | 6 | 3 |
